# A pragmatic controlled trial to improve the appropriate prescription of drugs in adult outpatients: design and rationale of the EDU.RE.DRUG study

**DOI:** 10.1017/S1463423620000249

**Published:** 2020-07-09

**Authors:** Manuela Casula, Enrica Menditto, Federica Galimberti, Veronica Russo, Elena Olmastroni, Lorenza Scotti, Valentina Orlando, Giovanni Corrao, Alberico L. Catapano, Elena Tragni

**Affiliations:** 1 Epidemiology and Preventive Pharmacology Service (SEFAP), Department of Pharmacological and Biomolecular Sciences, University of Milan, Milan, Italy; 2 IRCCS MultiMedica Hospital, Milan, Italy; 3 CIRFF, Center of Pharmacoeconomics, Federico II University of Naples, Naples, Italy; 4 Department of Statistics and Quantitative Methods (DiSMeQ), University of Milano-Bicocca, Milan, Italy

**Keywords:** appropriate prescription, educational intervention, pragmatic trial

## Abstract

**Introduction:**

Pharmacological intervention is an important component of patient care. However, drugs are often inappropriately used. It is necessary for countries to implement strategies to improve the rational use of drugs, including independent information for healthcare professionals and the public, which must be supported by well-trained staff. The primary objectives of the EDU.RE.DRUG (Effectiveness of informative and/or educational interventions aimed at improving the appropriate use of drugs designed for general practitioners and their patients) study are the retrospective evaluation of rates of appropriate prescribing indicators (APIs) and the assessment of the effectiveness of informative and/or educational interventions addressed to general practitioners (GPs) and their patients, aimed at improving prescribing quality and promoting proper drug use.

**Methods and analysis:**

This is a prospective, multicentre, open-label, parallel-arm, controlled, pragmatic trial directed to GPs and their patients in two Italian regions (Campania and Lombardy). The study data are retrieved from administrative databases (Demographic, Pharmacy-refill, and Hospitalization databases) containing healthcare information of all beneficiaries of the National Health Service in the Local Health Units (LHUs) involved. According to LHU, the GPs/patients will be assigned to one of the following four intervention arms: (1) intervention on GPs and patients; (2) intervention on GPs; (3) intervention on patients; and (4) no intervention (control). The intervention designed for GPs consists of reports regarding the status of their patients according to the APIs determined at baseline and in two on-line Continuous Medical Education (CME) courses. The intervention designed for patients consists in flyers and posters distributed in GPs ambulatories and community pharmacies, focusing on correct drug use.

A set of indicators (such as potential drug–drug interactions, unnecessary duplicate prescriptions, and inappropriate prescriptions in the elderly), adapted to the Italian setting, has been defined to determine inappropriate prescription at baseline and after the intervention phase. The primary outcome was a composite API.

**Ethics and dissemination:**

The study was approved by the Ethics Committee of the University of Milan on 7th June 2017 (code 15/17). The investigators will communicate trial results to stakeholders, collaborators, and participants via appropriate presentations and publications.

Registration details: NCT04030468. EudraCT number 2017-002622-21

## Strengths and limitations of this study

- This trial addresses a problem of great epidemiological, clinical, and socio-economic impact: the inappropriate prescription of drugs to adult patients in the outpatient setting.
- The definition of prescribing inappropriateness indicators adapted to the Italian context provides a useful tool both for the physician in the daily prescription activity and for the Local Health Units for the activities of evaluation and monitoring of the prescriptive performance.
- The use of existing data for baseline and outcome evaluation is a powerful and relative low-cost research tool that can be easily implemented on a larger scale. Despite this potential of prescription database analysis, a real measure of the appropriateness of prescriptions should be patient-based and evaluated by specialized personnel taking into consideration the characteristics of the patient.
- Since prescribing is uniquely managed by the doctor and is based on his/her final judgement, any intervention cannot impose decisions but only educate the doctor and support his/her activity; as a consequence, the integration of improved decision-making processes into the daily prescribing practice requires multidimensional interventions maintained over time.

## Rationale

Medicines are meant to improve health of patients; they do have, however, the potential to harm human subjects. The process of drug prescribing is therefore a fundamental component of the care of patients (World Health Organization, 2002). Appropriateness of prescribing is a balance of pharmacological rationality, the need of individual patients and economic aspects. It occurs when patients receive medications appropriate to their clinical needs, in doses that meet their own individual requirement, for an adequate time period and at the lowest cost to them and their community (World Health Organization, 2002). It can be defined as ‘the outcome of the process of decision making that maximizes net individual health gains within the society’s available resources’ (Buetow *et al*., 1997).

Optimization of drug prescribing has become an important public health issue worldwide (Hogerzeil, 1995; Dean *et al*., 2002; 2005). That is because evidence indicates high prevalence of inappropriate prescribing of medicines, especially in elderly people, which are characterized by chronic conditions and multimorbidity, leading to an increased use of drugs (polypharmacy). Inappropriate prescribing occurs, for example, when the physician prescribes an incorrect dosage and/or duration of treatment, drugs with significant drug–drug and drug–disease interactions, or fails to prescribe beneficial drugs (Spinewine *et al*., 2007). Notably, correct prescribing does not guarantee that drugs are used properly. Non-compliance to doctors’ prescriptions is very common (Casula *et al*., 2012). Therefore, patient involvement in the decision process could promote a conscious attitude, in compliance with the instructions received.

Inappropriate prescribing is associated with increased morbidity and mortality, increased cost of treatment, and decreased quality of life (Harrison *et al*., 2018). World Health Organization data show that more than 50% of all drugs are inappropriately prescribed or dispensed, and 50% of patients use them improperly (World Health Organization, 2002). Nearly 8% of medical examinations of patients with more than 65 years lead to the prescription of a Potentially Inappropriate Medication (PIM) (Goulding, 2004). Another European survey (Fialova *et al*., 2005) showed that 20% of elderly patients used at least 1 inappropriate medication, with substantial differences between Eastern Europe (41% in the Czech Republic) and Western Europe (range from 6% in Denmark to 27% in Italy). Using different definitions in various settings, observational studies showed that 21%–40% of patients have received at least one inappropriate medication (Liu and Christensen, 2002). In two cohort studies in Italy, 18% of elderly outpatients had one or more PIM prescriptions (Maio *et al*., 2006), and a substantial proportion of subjects was exposed to prescriptions at risk of potential drug–drug interaction (pDDI) (Tragni *et al*., 2013).

To limit the consequences of prescription of PIMs, improving rational use of drugs is a major focus to enhance quality and safety of care. It is thus necessary to implement a series of strategies, including information for healthcare professionals and the public from independent sources, which must be supported by well-trained staff (Hogerzeil, 1995). Different strategies have been developed and validated in this context (World Health Organization, 2002). Of note, interventions that included in-depth and updated education on drug therapy to physicians led to significant improvements in their performance/behaviour. Training and feedback control of prescribing should be associated with availability of on-line references for immediate identification and verification of potential erroneous prescribing (Thomas *et al*., 2008; Ostini *et al*., 2009). More generally, a good drug literacy allows more realistic perceptions and expectations, a shared medical decision making, and a responsible behaviour in using drugs. This approach seems promising and can be achieved through targeted campaigns of public education.

In this context, to measure inappropriateness is necessary to quantify the problem at baseline, to identify areas of concern which might require further review or development, and evaluate the effect of interventions (Donabedian, 1966). Moreover, the measure of inappropriateness in prescribing practice allows the physician to have a measure of his/her own performance, representing a point of comparison with colleagues within the same geographical area (Lovaglio, 2012) and a guide to intervene on critical situations of individual patients, besides having a general potential to raise physicians’ awareness about the topic. Given that prescribing is a mix of evidence-base for intervention with the drug, diagnosis, clinical judgement, and a certain element of clinical intuition, identifying objective measures of inappropriateness is extremely challenging (O’Connor *et al*., 2012b). Though, it is not surprising that the proposed indicators are umpteen, with different characteristics and potentials depending on the object of the measure and the context of application, and need to be updated and contextualized. Among them, the explicit criteria (O’Connor *et al*., 2012b; Masnoon *et al*., 2018; Curtin *et al*., 2019) are clearly defined statements, which highlight PIMs in particular clinical circumstances. They are mainly based on trial evidence, expert opinion, and consensus techniques (O’Connor *et al*., 2012a). The development of a simple, inexpensive, and time-efficient set of indicators which can be used routinely to evaluate prescribing practice and to assess the effectiveness of optimization strategies is therefore warranted.

In this context, the EDU.RE.DRUG (*Effectiveness of informative and/or educational interventions aimed at improving the appropriate use of drugs designed for general practitioners and their patients*) study has been designed to deeply investigate the prescribing practice among general practitioners (GPs) and the correct use of drugs by patients in two Italian regions and to assess the effectiveness of informative and/or educational interventions addressed to GPs and their patients, to improve prescribing quality, and to promote proper drug use.

## Methods and analysis

### Study design

EDU.RE.DRUG is a prospective, pragmatic, multicentre, open-label, parallel-arm, and controlled trial, started in April 2017 (see summary of study characteristics in Table S1, Supplementary material).

### Study setting and population

The clinical setting of the study is the general practice. The study population is composed by all GPs and all their adult patients aged ≥40 years from selected Local Health Units (LHUs) of two Italian regions, Lombardy and Campania.

### Data source and collection

In Italy, National Health Service (NHS) provides universal coverage largely free of charge at the point of delivery. The regions are responsible for organizing and delivering healthcare through the LHUs. The study data are retrieved from administrative databases containing healthcare data of all beneficiaries of the NHS in the LHUs involved: in 2017, about 2,800,000 beneficiaries for the 4 LHUs in Lombardy (Bergamo, Lecco, Mantova, and Monza Brianza) and 3,300,000 beneficiaries for the 4 LHUs in Campania (Avellino, Caserta, Napoli 1 Centro, and Napoli 2 Nord) (ISTAT, 1999). These databases, set up and constantly updated by regional or local health authorities, are:

- Demographic Databases, containing data on residents who receive NHS assistance (birth date and sex), and on GPs (birth date, sex, and number of patients).
- Pharmacy-refill Databases, containing data on drug prescriptions reimbursable by the NHS, including prescription date, dispensation date, the Anatomical Therapeutic Chemical (ATC) classification, marketing authorization code, number of boxes, and cost for NHS.
- Hospitalization Databases, containing data on hospital discharge at public or private hospitals of the regions, including the admission date, the primary and secondary diagnoses, and the date of discharge.

Compliance with national and European laws on personal data is guaranteed by LHUs through the generation of unique anonymous codes for each patient and each prescriber, providing guarantees in respect of the privacy of every citizen.

Data used in this project cover a time period ranging between the years 2014–2017 (baseline) and 2018–2019 (follow-up).

### Definition of performance indicators

For the evaluation of prescribing practice, patients in polytherapy were defined as having 5–9 or ≥10 single drugs prescribed in 1-year period.

Moreover, researchers selected some of the most commonly used drug classes (ACE-inhibitors [C09AA], angiotensin receptor blockers [C09CA], anti-asthmatics [R03], antibiotics [J01], proton pump inhibitors [A02BC], selective serotonin reuptake inhibitors [N06AB], serotonin-norepinephrine reuptake inhibitor [N06AX], and statins [C10AA]) to be described as percentage of patients on each treatment and as amount of defined daily doses (DDD) prescribed per 1000 inhabitants/die.

### Definition of inappropriate prescribing indicators

The research team reviewed the scientific literature on the topic and identified a set of indicators that had to:

– be explicit indicators that require each prescription to be compared with a set of pre-defined standards, within the context of the individual patient;
– be applicable and valid regardless of the patient’s clinical characteristics;
– include only drugs available on Italian market and reimbursed by Italian NHS (which are therefore traced into administrative databases).

Prescription of pDDIs has been defined based on *MediRisk software*, developed by Medilogy group, based on INXBASE by Medbase, a Finnish company formed by experts in pharmacotherapy, which produces medical decision support databases to safeguard effective and safe use of drugs. INXBASE is a drug–drug interaction database containing short and concise evidence-based information concerning consequences of and recommendations for more than 20,000 drug interactions (Inxbase; https://www.medbase.fi/en/professionals/inxbase/). Interactions are classified according to clinical significance (from minor ‘A’ to contraindicated ‘D’) and documentation level (from ‘evidence from in vitro studies’ ‘0’ to ‘evidence from randomised clinical trials, systematic reviews, or meta-analyses’ ‘4’). In this project, two drugs were considered potentially interacting if their coverage periods (calculated since dispensation date and based on DDDs) overlapped of at least 1 day. Only pDDIs with major ‘C’ clinical significance excluded those with level of documentation ‘0’, and contraindicated ‘D’ clinical significance was considered.

Therapeutic duplicates (TDs) have been defined as two or more prescribed drugs from the same chemical subgroup (same ATC code at the fourth level but different ATC code at the fifth level) (Fulda *et al*., 2004) with at most 3 days between the two dispensation dates.

Only in the elderly population (aged ≥65 years), we defined the ERD-list (EDU.RE.DRUG list, Table S2 in Supplementary material) developed based on the updated Beers criteria (Radcliff *et al*., 2015), the STOPP&START criteria (O’ Mahony *et al*., 2018), and the EU-(7)-PIM list (Renom-Guiteras *et al*., 2015). The three lists were merged and adapted to Italian setting by selecting only drugs available on the Italian market and reimbursed by Italian NHS. Moreover, the selection was limited to drugs always to be avoided in elderly patients, excluding drugs that should be used with caution or avoided in certain patients with certain diseases or conditions, as these circumstances cannot be evaluated through administrative databases.

The appropriate prescribing indicators (APIs) in elderly comprised also high scores (≥3) of the Anticholinergic Cognitive Burden (ACB) scale (Boustani *et al*., 2008) and of the Sedative Load (SL) score (Linjakumpu *et al*., 2003) from the published lists, again selecting only drugs available on the Italian market and reimbursed by Italian NHS.

### Definition of appropriate use indicator

For each medication, adherence rate will be assessed for the following chronic therapies [ATC]:

- antidiabetics [A10B]
- anti-hypertensive drugs [C02, C03, C07, C08, C09]
- lipid-lowering drugs [C10A]
- anti-osteoporosis drug [M05BA, M05B].

Adherence will be measured through the proportion of days covered (PDC) calculation (Pednekar *et al*., 2019). PDC is defined as the number of days covered by medication divided by the total number of days in follow-up. For each prescription, the coverage will be calculated as total amount of drug divided by the specific DDD. PDC ranges from 0 to 1, with 1 corresponding to 100% medication adherence.

### Study intervention

**Figure 1.**
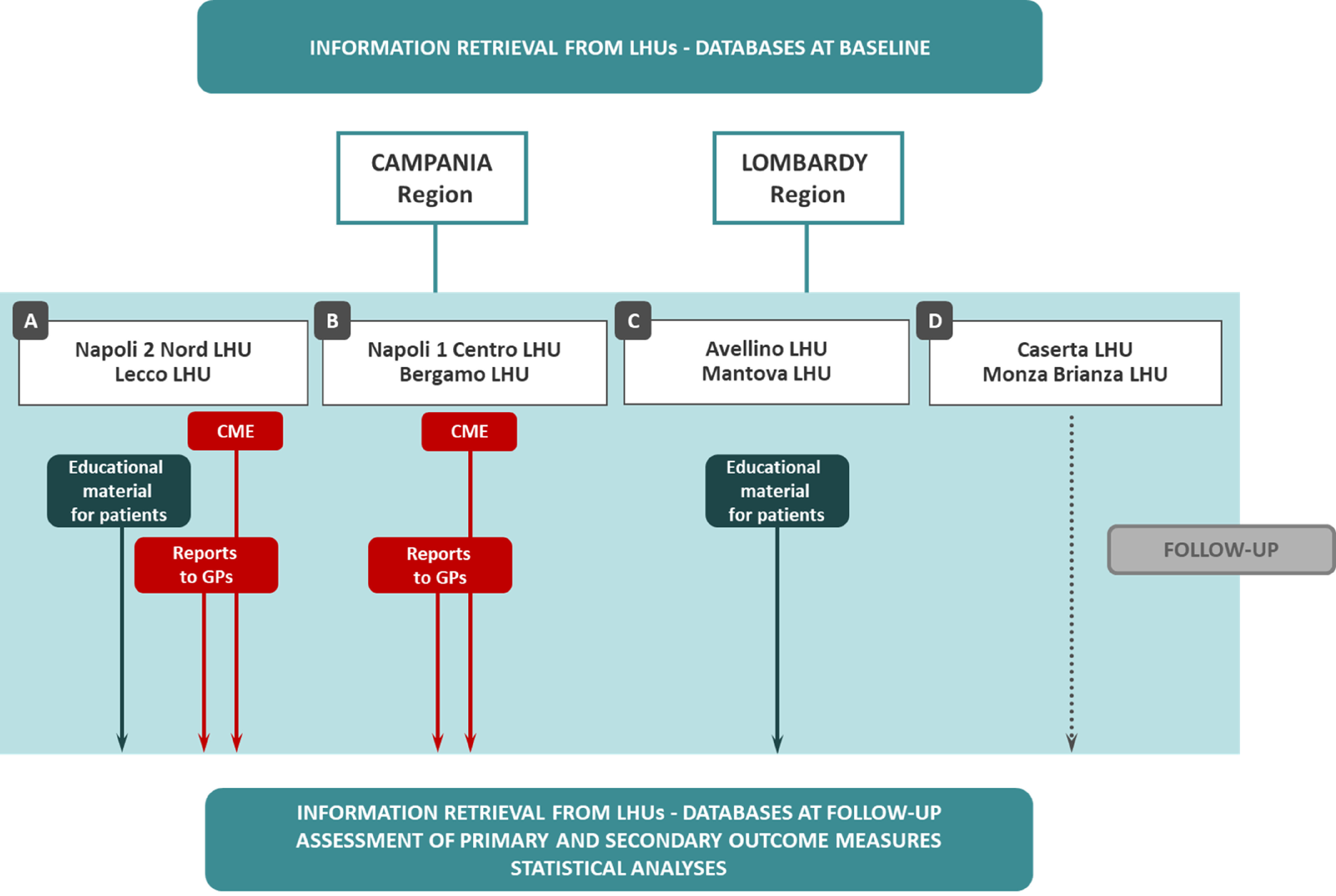
Design of the study and description of the four study groups

The GPs and their patients have been assigned to one of the following arms (Figure 1):

A: intervention on GPs and patients (LHUs of Napoli 2 Nord and Lecco);
B: intervention on GPs (LHUs of Napoli 1 Centro and Bergamo);
C: intervention on patients (LHUs of Avellino and Mantova);
D: control group (LHUs of Caserta and Monza Brianza).

The intervention addressing GPs consists in:

- feedback reports, describing inappropriate prescription status (prevalence of each pre-defined indicator of performance and of inappropriate prescribing, as listed above, evaluated in 2016) of their patients in comparison to median levels of each own LHU.
- two free on-line CME courses about rational prescribing and appropriateness measurement. The first course was focused on the presentation of the project and on general aspects concerning the inappropriate prescribing in general practice and the categories of the most vulnerable patients, such as the elderly or poly-treated patients, with the presentation of clinical cases of possible inappropriate prescriptions. The second course concerned the measurement of APIs, the guided reading of reports, and recommendations for prescribing to the complex patient and for medication review.

Notably, participation to CME courses was not mandatory, as well as both the courses and the reports received at baseline would not necessarily lead to changes in GPs’ prescriptive behaviour.

The intervention designed for patients consists in flyers and posters distributed in GPs’ ambulatories and community pharmacies, focusing on correct drug use (efficacy/safety, adherence to GP indications, and self-medication).

Time frame dedicated to the sending of feedback reports and to the delivery of educational material was January–March 2018.

### Study outcome

The study outcome was a composite outcome of API of ERD list, pDDIs, and TDs. The primary end point was therefore defined as the variation of the median prevalence of the composite API after the intervention in the groups with the intervention on GPs (A+B arms) compared to baseline. The prevalence will be calculated at GP’s individual level as the ratio between subjects with the composite API and total GP’s over-40-year subjects.

The secondary end points comprised the (1) variation of the median prevalence of each single API and performance indicator after the intervention, (2) evaluation of difference in efficacy among different types of intervention, (3) identification of predictors of poor prescription appropriateness, (4) health technology assessment (HTA) of intervention implemented, and (5) level of GP satisfaction assessed through an ad hoc web-based questionnaire.

### Sample size and statistical analysis

The study design is a non-randomized, open-label, cluster intervention. All experimental units (GPs and/or patients) in each cluster receive the scheduled treatment. Assuming that at least 40% of resident in the involved LHUs of the two regions (1.1 million in Lombardy and 1.3 million in Campania) receive at least one prescription during 1-year period, considering a type I error of 5%, a power of 80% would allow to detect, at LHU level, a difference in the reduction of inappropriateness prevalence of at least 5% between intervention and control group.

#### Analysis of indicators

The indicators of performance and of inappropriate prescribing will be determined at baseline and after the intervention, by measuring the explicit indicators defined above within each GP on 1-year base. The unit of analysis will be the patient or his/her prescriptions, depending on the indicator, and GP identified as the clustering factor within each indicator will be examined.

The performance indicators will be evaluated separately on the subpopulations of 40–64 years and ≥65 years. Polytherapy will be evaluated as the percentage of patients with 5–9 drugs or with ≥10 drugs on total GP’s subjects in each age class. Prescription of selected drug classes (as listed above) will be evaluated as percentage of patients on each treatment on total GP’s subjects in each age class and as amount of DDD prescribed per 1000 inhabitants/die in each age class.

Regarding APIs, pDDIs and TDs will be evaluated on subjects ≥40 years old, while drugs in EDR list, ACB scale, and the SL score will be evaluated on subjects ≥65 years old. For each API, the percentage of patients with at least one prescription meeting the API criteria on total GP’s subjects in the specific age class will be determined.

Adherence will be calculated by selecting all patients with a first prescription for the medication of interest within 1-year period. Patients will be required not to have prior prescription of that drug in the year before the index date (defined as the date of the first prescription fill in the period for the selected therapy), to select only incident users. Patients will also be required to have 1 year of enrolment after the index date to allow complete adherence evaluation at 1 year of follow-up.

#### Identification of determinants of inappropriate prescribing

A multilevel model will be considered to identify the association between several variables (related to patients, physicians, or LHUs) and the composite API. The model will allow to take into account the hierarchical structure of the data, with patients nested within physicians and physicians nested within LHU. The considered potential determinants will be measured at the patient level (age, sex, clinical profile using the Chronic Disease Score (Vonkorff *et al*., 1992), and number of prescriptions received), at the physician level (age, sex, and number of patients assisted), and at the LHU level (inhabitants, population density, and number of GPs per 1000 inhabitants).

#### Interventions effectiveness (pre-post analysis)

The primary and secondary outcomes will be evaluated in a 6-month period before intervention (pre-intervention phase, October 2016–March 2017) and in a 6-month period after the intervention (post-intervention phase, October 2018–March 2019). Depending on LHU and on type of data, administrative data usually require 3–6 months to be processed and made available. The difference (Δ pre-post) in the outcomes will be estimated separately for each LHU. Appropriate contrasts to compare Δ in the different groups of intervention and the corresponding confidence intervals will be estimated. The standard error for each contrast will be assessed by an appropriate normality assumption or, if this assumption is not plausible, by other methods such as the nonparametric bootstrap.

Since the study is not randomized, to consider the potential confounding due to physicians’ and patient’s characteristics, two additional analyses will be made at physician and patient level. Firstly, the Δ of each physician will be evaluated. A linear mixed regression model will be applied including the Δ as response variable and physicians and the LHU characteristics as well as the type of intervention as covariates. To take into account that physicians are clustered within LHU, a random effect for LHU will be considered. A generalized linear mixed regression model considering post-intervention prevalence of composite API as response variable will be estimated. In this model, patient-, physician-, or LHU-level covariates will be included. Two random effects will be considered in the model: one for the physician and one for the LHU. To take into account the baseline probability of being inappropriately treated, we will include in the model the prevalence of composite API for the patient’s physician evaluated before the intervention.

To evaluate the effects of interventions in terms of the subsequent mortality risk, all patients receiving at least one prescription during the post-phase will be selected and followed for 1 year. The vital status of each patient will be recorded during the year. A Cox proportional hazard regression model will be applied to evaluate the association between the intervention and mortality. The response variable will be the time to death and the model will include different covariates measured at the patient-, physician-, and LHU-level, the type of intervention, and the prevalence of composite API for the physician assigned to each patient as evaluated before the intervention.

#### Health technology assessment

A HTA of the intervention will be performed by using the following typical approaches: (Husereau *et al*., 2013):

– systematic review of the literature, in order to define the status of our interventions
– efficacy and effectiveness
– social, legal, political, and ethical impacts
– cost and economic evaluation.

Total expenditure for all PIMs will be calculated. Costing information may consist of actual costs, prices, or tariffs, as appropriate. The cost analysis will be performed from the third party payer (NHS) perspective. Costs will be calculated as the Net Ingredient Cost of the dispensed drug and the total expenditure, which will include the pharmacist dispensing fee where appropriate.

#### Evaluation of GPs satisfaction

An ad hoc questionnaire will be administrated to GPs in anonymous web form, in order to detect their satisfaction about the intervention. It will be structured into questions focused on:

1. opinion on the utility/efficacy of CME courses
2. opinion on the utility/efficacy of feedback prescription reports
3. impact on professional practice.

The frequency of degree of satisfaction will be determined for each response.

### Patient and public involvement

This research will be done without patient involvement. Patients will be not invited to comment on the study design and not consulted to develop patient relevant outcomes. Patients will be not invited to contribute to the writing or editing of this document for readability or accuracy.

## Expected results and impact

In Italy, there are no official policy statements or regulatory guidelines on management of inappropriate prescribing. However, there is evidence of a growing awareness of the problem (Mannucci *et al*., 2014; Franchi *et al*., 2016). Relatively, few trials have focused on interventions to improve appropriate prescribing in primary care. Importantly, in Italy GPs have a key role in drug prescribing, in summarizing pharmacological recommendations from different specialists, and in implementing the therapeutic reconciliation after a hospital discharge. Thus, they are the obvious target of an intervention aimed to optimize drug management.

We expect the intervention to improve prescribing practice, reducing instances of inappropriate behaviour. This effect would be guided possibly both by delivering specific training on this topic (ECM course) and by making GPs aware of the levels of APIs estimated on their patients and on the LHU. Indeed, the report will provide each GP with the situation of his/her prescribing practice, highlighting their own inappropriate situations, and the comparison with colleagues who work in the same area. Benchmarking is a strong driver for quality improvement and has been shown to increase competitive standards, resulting in an overall increase of the performance (Ettorchi-Tardy *et al*., 2012).

Establishing the impact of this pragmatic design on medical practice is difficult, especially because the interventions will not necessarily influence GPs’ prescribing behaviour nor result in any therapy change. However, previous studies showed that interventions targeting individual professional, such as feedback and academic detailing, could be effective in improving prescribing practice. Results from a Cochrane systematic review involving 69 studies and more than 15,000 health professionals reported an increase (+5.6%) in compliance with desired practice (O’Brien *et al*., 2007). Additionally, in another pragmatic trial conducted in Italy, a similar intervention addressed to GPs has been shown to improve adherence to therapy, suggesting a benefit also for the patient’s attitude and for the patient–physician relationship (Casula *et al*., 2016).

This study, conducted through a retrospective evaluation on administrative databases of drug prescriptions and hospitalizations, will allow to explore different patterns of prescribing in real-world setting and to analyse the complexity of drug prescription, highlighting possible dangerous prescribing habits. The definition of indicators to describe inappropriate prescription and identify patients at higher risk of medicine-related problems based on Italian drug-utilization patterns will provide tools specifically tailored to the Italian context but also adaptable to other national contexts. Moreover, data from EDU.RE.DRUG study will be used to identify predictors of inappropriate prescribing and therapeutic areas most affected by this problem, in order to establish priorities for actions, to focus efforts, and optimize the scarce available resources.

## Ethics

The protocol has been registered in ClinicalTrials.gov (identifier NCT04030468) and in EU Clinical Trials Register (identifier: EudraCT 2017-002622-21).

The study was approved by the Ethics Committee of the University of Milan on 07 June 2017 (code 15/17).

Procedures aimed at protecting personal data will be implemented in order to safeguard privacy and to prevent the identification of individual data (according to Italian law D.Lgs. n. 196/2003). Anonymized regional administrative data can be used without a specific written informed consent when patient information is collected for healthcare management and healthcare quality evaluation and improvement (according to art. 110 on medical and biomedical and epidemiological research, Legislation Decree 101/2018).

## Dissemination

A variety of methods will be used to ensure the maximum visibility for the project and its results. Publication of our study protocol provides an important first step towards this direction. Moreover, the description of the study and the material for patients have been made available on the website (http://www.sefap.it/web/ms/index.php?idms=11, in Italian language), as well as its main results will be published here.

Similarly, the study results, given their applicability and implications for the general population, will be disseminated in investigator meetings and in articles published in scientific journals.

## Supplementary material

For supplementary material accompanying this paper visit http://dx.doi.org/10.1017/S1463423620000249. click here to view supplementary material

## Appendix EDU.RE.DRUG Group

Alberico L Catapano^1,2^, Elena Tragni^1^, Enrica Menditto^3^, Giovanni Corrao^4^, Manuela Casula^1,2^, Federica Galimberti^1^, Elena Olmastroni^1^, Veronica Russo^3^, Valentina Orlando^3^, Lorenza Scotti^4^, Antonella Zambon^4^, Marco Gambera^5^, Rossana Piccinelli^5^, Samanta Sonzogni^5^, Valter Valsecchi^6^, Eugenio Scopinaro^6^, Sandro Raineri^7^, Alessia Speziali^7^, Simona Creazzola^8^, Michele Tari^9^, Mariano Fusco^10^

^1^Epidemiology and Preventive Pharmacology Service (SEFAP), Department of Pharmacological and Biomolecular Sciences, University of Milan, Milan, Italy

^2^IRCCS MultiMedica Hospital, Sesto S. Giovanni, Milan, Italy

^3^CIRFF, Center of Pharmacoeconomics, Federico II University of Naples, Naples, Italy

^4^Department of Statistics and Quantitative Methods (DiSMeQ), University of Milano-Bicocca, Milan, Italy

^5^Bergamo Local Health Authority, Bergamo, Italy

^6^Brianza Local Health Authority, Lecco, Italy

^7^Mantova Local Health Authority, Mantova, Italy

^8^Local Health Authority Napoli1 Centro, Naples, Italy

^9^Local Health Authority Caserta, Caserta, Italy

^10^Local Health Authority Napoli2 Nord, Frattamaggiore (NA), Italy
