# Supplementary material for: A pragmatic controlled trial to improve the appropriate prescription of drugs in adult outpatients: design and rationale of the EDU.RE.DRUG study
Source: Prim Health Care Res Dev. 2020 Jul 9;21:e23. doi: 10.1017/S1463423620000249 (PMC7372175; doi:10.1017/S1463423620000249)
Supplement: Supplementary file 1 [file S1463423620000249sup001.docx]

**A pragmatic controlled trial to improve the appropriate prescription of drugs in adult outpatients: design and rationale of the EDU.RE.DRUG study**

**Running Title: The EDU.RE.DRUG trial protocol**

**Supplementary Material**

**Table S1** Summary table explaining fundamental characteristics of the EDU.RE.DRUG trial

| Study design | Pragmatic, prospective, multicentre, open-label, parallel-arm, controlled trial. |
| --- | --- |
| Study population | All adult patients (aged ≥40 years) registered with a GP from eight LHUs of Lombardy) and Campania, and with at least one drug prescription in the years considered (as tracked by the administrative databases containing healthcare data). |
| Intervention arms | Four arms of intervention:  A: intervention on GPs and patients (LHUs of Napoli 2 Nord and Lecco);  B: intervention on GPs (LHUs of Napoli 1 Centro and Bergamo);  C: intervention on patients (LHUs of Avellino and Mantova);  D: control group (LHUs of Caserta and Monza Brianza). |
| Intervention definition | - Intervention on GPs: distribution of feedback reports, describing inappropriate prescription status of their patients in comparison to LHU’s median levels and administration of two free on‐line Continuous Medical Education (CME) courses about rational prescribing and drug appropriateness measurement.  - Intervention on patients: distribution of flyers and posters in GPs' ambulatories and community pharmacies, focusing on proper drug use. |
| Primary outcome definition and measure | Composite of three inappropriate prescribing indicators (API): ERD-list (prescription of drugs to be avoided in elderly patients), pDDIs (prescription of potential interacting drugs) and TDs (prescription of duplicates).  Variation of the median prevalence of the composite API after the intervention in the groups with the intervention on GPs (A+B arms) compare to baseline. The prevalence of the primary outcome will be calculated at GP’s individual level, as the ratio between the number of subjects experiencing the composite API and the total number of subjects. |

**Table S2** ERD-lists for assessment of inappropriate prescribing among elderly

| **ATC code** | **Drug Name** | **Reason for PIM** |
| --- | --- | --- |
| A02BC*, B01AC56 | Proton pump inhibitors (>8 weeks) | Long-term high dose therapy is associated with an increased risk of C. difficile infection and hip fracture. Inappropriate if used >8 weeks in maximal dose without clear indication |
| A10AB01, A10AB04, A10AB05, A10AB06 | Insulin, sliding scale (without concomitant treatment with basal insulin) | No benefits demonstrated in using sliding-scale insulin. Might facilitate fluctuations in glycemic levels |
| A10BB01, A10BD02, A10BB07, A10BB12, A10BD06 | Glibenclamide, Glipizide, Glimepiride | Risk of protracted hypoglycemia |
| A10BF01 | Acarbose | No proven efficacy |
| A10BG03, A10BD05, A10BD06, A10BD09 | Pioglitazone | Age-related risks include bladder cancer, fractures and heart failure. Use for more than one year has been associated with an increased risk of bladder cancer. May increase the incidence of fractures of the upper arms, hands and feet in female diabetics (compared to other oral antidiabetic agents). Can cause fluid retention in older adults, which may exacerbate or precipitate heart failure |
| B01AA07 | Acenocoumarol | Risk of bleeding, especially in people with difficult control of INR value |
| B01AC05 | Ticlopidine | Risk of altered blood counts |
| C01AA08 | Metildigoxin | Elevated glycoside sensitivity in older adults (women>men); risk of intoxication |
| C01BA03 | Disopyramide | Potent negative inotrope; anticholinergic side effects; may induce heart failure; may cause sudden cardiac death. Data suggest that for most older adults rate control yields better balance of benefits and harms than rhythm control |
| C01BC03 | Propafenone | High risk of drug interactions. Data suggest that for most older adults rate control yields better balance of benefits and harms than rhythm control |
| C01BC04 | Flecainide | Higher rate of adverse effects, especially in older adults. Data suggest that for most older adults rate control yields better balance of benefits and harms than rhythm control |
| C02AB01 | Methyldopa | Risk of orthostatic hypotension, bradycardia, syncope, CNS side effects (sedation, depression, cognitive impairment) |
| C02AC05 | Moxonidine | Risk of orthostatic hypotension, bradycardia, syncope, CNS side effects (sedation, depression, cognitive impairment) |
| C08CA05 | Nifedipine | Increased risk of hypotension; myocardial infarction; increased mortality |
| G02CB03 | Cabergoline | CNS side effects |
| G03BA03 | Testosterone | Potentialforcardiacproblem |
| G03AA09, G03AA10, G03AB06, G03CA01, G03CA03, G03CA04, G03CA09, G03CX01, G03FA01, G03FA11, G03FA14, G03FA17, G03FB05, G03FB08, G03FB09, G03FB12, L02AB01 | Ethinylestradiol, Estradiol, Promestriene, Tibolone, Megestrol | Evidence for carcinogenic potential (breast and endometrial cancer) and lack of cardioprotective effect in older women |
| H01BA02 | Desmopressin | High risk of hyponatremia |
| M01AB01 | Indometacin | Very high risk of GI bleeding, ulceration, or perforation, which may be fatal; risk of CNS disturbances |
| M01AB05, M01AB16 | Diclofenac, Aceclofenac | Very high risk of GI bleeding, ulceration, or perforation, which may be fatal; cardiovascular contraindications |
| M01AB15 | Ketorolac | Very high risk of GI bleeding, ulceration, or perforation, which may be fatal |
| M01AC01, M01AC06 | Piroxicam, Meloxicam | Very high risk of GI bleeding, ulceration, or perforation, which may be fatal |
| M01AC05 | Lornoxicam | Very high risk of GI bleeding, ulceration, or perforation, which may be fatal; cardiovascular contraindications |
| M01AE03 | Ketoprofen | Very high risk of GI bleeding, ulceration, or perforation, which may be fatal |
| M01AE09 | Flurbiprofen | Very high risk of GI bleeding, ulceration, or perforation, which may be fatal; cardiovascular contraindications |
| M01AX01 | Nabumetone | Very high risk of GI bleeding, ulceration, or perforation, which may be fatal; cardiovascular contraindications |
| N02AD01 | Pentazocine | Risk of delirium and agitation |
| N02AX02 | Tramadol | More adverse effects in older adults; CNS side effects such as confusion, vertigo and nausea |
| N03AA02 | Phenobarbital | Risk of sedation, paradoxical excitation High rate of physical dependence, tolerance to sleep benefits, greater risk of overdose at low dosages |
| N03AB02 | Phenytoin | Narrow therapeutic window; increased risk of toxicity in older adults (e.g. CNS and hematologic toxicity) |
| N03AE01 | Clonazepam | Risk of falls, paradoxical reactions |
| N03AX11 | Topiramate | Risk of cognitive-related dysfunction (e.g., confusion, psychomotor slowing) |
| N04AA01, N04AA02, N04AB02 | Trihexyphenidyl, Biperiden, Orphenadrine | Risk of anticholinergic and CNS side effects including orthostatic hypotension, falls, sedation, weakness, confusion, amnesia |
| N04BC01 | Bromocriptine | Risk of CNS side effects |
| N05AC01 | Propericiazine | Anticholinergic and extrapyramidal side effects (tardive dyskinesia); parkinsonism; hypotonia; sedation; risk of falling; increased mortality in persons with dementia |
| N06AA02, N06AA04, N06AA06, N06AA09, N06AA10 | Imipramine, Clomipramine, Trimipramine, Amitriptyline, Nortriptyline | Peripheral anticholinergic side effects (e.g. constipation, dry mouth, orthostatic hypotension, cardiac arrhythmia); central anticholinergic side effects (drowsiness, inner unrest, confusion, other types of delirium); cognitive deficit; increased risk of falling |
| N06AB03 | Fluoxetine | CNS side effects (nausea, insomnia, dizziness, confusion); hyponatremia |
| N06AB05 | Paroxetine | Higher risk of all-cause mortality, higher risk of seizures, falls and fractures. Anticholinergic adverse effects |
| N06AB08 | Fluvoxamine | Higher risk of all-cause mortality, self-harm, falls, fractures and hyponatraemia |
| N06BA04 | Methylphenidat | May cause or worsen insomnia; concern due to CNS-altering effects; concern due to appetite-supressing effects |
| R06AD02 | Promethazine | Anticholinergic side effects (e.g. confusion, sedation) |

INR international normalized ratio; CNS central nervous system; GI gastrointestinal
